# Dysregulation of ferroptosis-related genes in granulosa cells associates with impaired oocyte quality in polycystic ovary syndrome

**DOI:** 10.3389/fendo.2024.1346842

**Published:** 2024-02-06

**Authors:** Jialyu Huang, Hancheng Fan, Chenxi Li, Kangping Yang, Chaoyi Xiong, Siyi Xiong, Shenghui Feng, Shen Chen, Bangqi Wang, Yufang Su, Boyun Xu, Haiyan Yang, Ni Wang, Jing Zhu

**Affiliations:** ^1^ Center for Reproductive Medicine, Jiangxi Maternal and Child Health Hospital, National Clinical Research Center for Obstetrics and Gynecology, Nanchang Medical College, Nanchang, China; ^2^ Department of Histology and Embryology, School of Basic Medicine, Nanchang University, Nanchang, China; ^3^ The Second Clinical Medical College of Nanchang University, The Second Affiliated Hospital of Nanchang University, Nanchang, China; ^4^ Department of Pathology, Jiangxi Maternal and Child Health Hospital, National Clinical Research Center for Obstetrics and Gynecology, Nanchang Medical College, Nanchang, China; ^5^ Department of Clinical Medicine, School of Queen Mary, Nanchang University, Nanchang, China; ^6^ Department of Oncology, Jiangxi Maternal and Child Health Hospital, National Clinical Research Center for Obstetrics and Gynecology, Nanchang Medical College, Nanchang, China; ^7^ Center for Reproductive Medicine, The First Affiliated Hospital of Wenzhou Medical University, Wenzhou, China; ^8^ Department of Anesthesiology, Xi’an Children’s Hospital, Xi’an, China

**Keywords:** polycystic ovary syndrome, ferroptosis, granulosa cell, oocyte quality, *in vitro* fertilization

## Abstract

**Background:**

Poor oocyte quality remains one of the major challenges for polycystic ovary syndrome (PCOS) patients during *in vitro* fertilization (IVF) treatment. Granulosa cells (GCs) in PCOS display altered functions and could cause an unfavorable microenvironment for oocyte growth and maturation. Ferroptosis is a new form of programmed cell death, but its role in PCOS has been largely unclarified.

**Methods:**

Ferroptosis-related differentially expressed genes (DEGs) of GCs in women with PCOS were identified by bioinformatic analyses of GSE155489 and GSE168404 datasets. Functional enrichment analyses were conducted using Gene Ontology and Kyoto Encyclopedia of Genes and Genomes. Core ferroptosis-related genes were further screened by random forest, and evaluated for diagnostic value by receiver operating characteristic curve analyses. Gene expression was validated by real-time quantitative polymerase chain reaction of collected GC samples, and analyzed for association with oocyte quality. In addition, gene regulatory network was constructed based on predicted RNA interactions and transcription factors, while potential therapeutic compounds were screened through molecular docking with crystallographic protein structures.

**Results:**

A total of 14 ferroptosis-related DEGs were identified. These DEGs were mainly enriched in reactive oxygen species metabolic process, mitochondrial outer membrane, antioxidant activity as well as ferroptosis and adipocytokine signaling pathways. Eight core ferroptosis-related genes (ATF3, BNIP3, DDIT4, LPIN1, NOS2, NQO1, SLC2A1 and SLC2A6) were further selected in random forest model, which showed high diagnostic performance for PCOS. Seven of them were validated in GC samples, and five were found to be significantly and positively correlated with one or more oocyte quality parameters in PCOS patients, including oocyte retrieval rate, mature oocyte rate, normal fertilization rate, and good-quality embryo rate. Gene regulatory network revealed JUN and HMGA1 as two important transcription factors, while dicoumarol and flavin adenine dinucleotide were predicted as small molecules with therapeutic potential.

**Conclusions:**

This is the first comprehensive report to study the differential expression of ferroptosis-related genes in GCs of PCOS and their clinical relevance with oocyte quality. Our findings could provide novel insights on the potential role of GC ferroptosis in PCOS pathogenesis, diagnosis, and targeted treatment.

## Background

Polycystic ovary syndrome (PCOS), a common endocrine disorder, affects 5-20% of reproductive-aged women worldwide and accounts for approximately 80% of anovulatory infertility (1). The condition is characterized by a heterogeneous clustering of androgen excess, ovulatory dysfunction, polycystic ovarian morphology, as well as metabolic abnormalities including obesity and insulin resistance (2). During *in vitro* fertilization (IVF) treatment, women with PCOS have typically increased oocyte yield from stimulation, while the maturation, fertilization, and implantation rates are decreased due to poor oocyte quality (3–6).

As the most abundant cells in ovary, granulosa cells (GCs) surround and bi-directionally interact with oocytes via paracrine signals and gap junctions (7, 8). Both types of GCs, mural and cumulus, coordinate to play essential roles in normal steroidogenesis and folliculogenesis (9, 10). Previous studies have shown that GCs in PCOS displayed altered proliferation, apoptosis, autophagy, mitochondrial function and inflammatory response, consequently leading to an unfavorable microenvironment for oocyte growth and maturation (11–15). Therefore, investigating the dysfunction of GCs and its underlying mechanisms could be scientifically and clinically crucial to improve oocyte quality of PCOS patients.

Ferroptosis is a new form of programmed cell death that mainly relies on iron accumulation, lipid peroxidation, and subsequent plasma membrane damage (16). Due to the role of iron in mediating enzyme activity and production of reactive oxygen species (ROS), ferroptosis is strictly regulated by iron metabolism involving iron uptake, storage, utilization, and efflux (17). Mounting evidences have documented excessive or deficient ferroptosis in a plethora of human diseases and proposed novel targets for pharmacological therapy (16). In PCOS women, mild iron overload has been observed possibly due to the iron sparing effect of menstrual irregularity and decreased hepcidin secretion facilitating iron absorption (18, 19). By analyzing peripheral blood CD4+ T cells of PCOS patients, Nasri et al. (20) identified several differentially expressed proteins that were enriched in ferroptosis pathway. In PCOS-model rats, Zhang et al. (21) also found increased gravid uterine and placental ferroptosis, which could be suppressed by antioxidant N-acetylcysteine (22). Recently, several *in vitro* studies have revealed a relationship between ferroptosis and GCs in PCOS (11, 23, 24), while the comprehensive regulatory network is still poorly defined and the clinical relevance remains largely unclear.

Using RNA sequencing data from publicly available Gene Expression Omnibus (GEO) database, we designed this *in silico* study to explore the expression of ferroptosis-related genes in GCs of PCOS. Moreover, we evaluated the correlation of core differential genes with oocyte quality in PCOS patients undergoing IVF cycles. Our study should provide novel insights on the pathogenesis, diagnosis and treatment of PCOS.

## Methods

### Dataset acquisition

Two RNA-seq datasets GSE155489 and GSE168404 were downloaded from the GEO database (https://www.ncbi.nlm.nih.gov/geo/). The dataset GSE155489 was performed on the GPL20795 (HiSeq X Ten) platform and included GC samples from 4 PCOS patients and 4 matched controls (25), while GSE168404 based on GPL1679 (Illumina HiSeq 2500) was made of GC samples from 5 PCOS patients and 5 matched controls (26).

### Differential expression analysis

The *limma* package in R software (version 3.6.1) was used to identify differentially expressed genes (DEGs), with adjusted *P*<0.05 and absolute fold change (|FC|)>1.5 determined as the thresholds. Volcano plots by *ggplot2* package and hierarchical clustering heatmaps by *pheatmap* package were employed for visualization of DEGs.

A total of 259 ferroptosis-related genes (FDGs) were obtained from the FerrDb database (http://www.zhounan.org/ferrdb/index.html), including 108 drivers, 69 suppressors and 111 markers (27). In addition, we assembled another 173 FDGs through published literatures (28, 29) and the merged set contained 291 FDGs after deduplication (**Supplementary Table S1**). Overlapping genes between DEGs and FDGs were selected for further analysis using the *VennDiagram* package.

### Functional enrichment analysis

To further assess the biological processes (BP), cellular components (CC), molecular functions (MF) and pathways involved in the DEGs associated with ferroptosis, Gene Ontology (GO) and Kyoto Encyclopedia of Genes and Genomes (KEGG) analyses were conducted using the *clusterpProfiler* package in R software. The results were considered as significantly enriched with *P*<0.05.

### Identification of core genes by random forest

Random forest is a supervised machine learning algorithm that combines multiple decision trees. It not only aggregates bootstrap samples to build each of the classification trees, but also randomly utilizes a certain percentage of all features for more accurate estimation and prediction. Therefore, random forest can provide a powerful ranking method to select core genes in diseases (30). To further filter out ferroptosis-related DEGs of low importance, we used the *randomForest* package in R software according to the criteria of Mean Decrease Gini (MDG) ≥0.05. Receiver operating characteristic (ROC) curve analyses were performed with the *pROC* package to evaluate the diagnostic value of retaining genes, quantified by the area under the curve (AUC) in both datasets.

### Regulatory network construction

ENCORI (https://starbase.sysu.edu.cn/) is an open-source platform for identifying the RNA interactions from multi-dimensional sequencing data, from which we obtained the interacted messenger RNAs (mRNAs), microRNAs (miRNAs) and long non-coding RNAs (lncRNAs) of core ferroptosis-related DEGs (31). In addition, the transcription factors (TFs) corresponding to core genes were extracted from TRRUST v2 (https://www.grnpedia.org/trrust), a comprehensive reference database of transcriptional regulatory interactions based on text mining and manual curation (32). A multi-factor network was constructed and visualized using the Cytoscape software (version 3.9.1).

### Molecular docking

To predict small therapeutic molecules targeting GC ferroptosis in PCOS, structures of core proteins were retrieved from Protein Data Bank (https://www.rcsb.org/) (33), while molecular information of available compounds were downloaded from DrugBank (https://go.drugbank.com/) (34). Molecular docking was carried out by AutoDock Vina software (version 1.2.0). Potential compounds were screened according to binding affinity ≤-7 kcal/mol, and the results were visualized using PyMOL software (version 2.5.2).

### Clinical sample collection

Ovarian GCs were collected from patients who underwent IVF treatment with gonadotropin-releasing hormone antagonist (GnRH-ant) protocol at the Center for Reproductive Medicine, the First Affiliated Hospital of Wenzhou Medical University. Ten PCOS patients were enrolled according to the revised 2003 Rotterdam consensus criteria (35), while the control group included 8 non-PCOS patients with matched age and body mass index, regular ovulatory cycle, and infertility caused by tubal or male factors. Women were excluded in cases of advanced reproductive age (≥38 years), diminished ovarian reserve, endometriosis, ovarian surgery history, chromosomal abnormalities, autoimmune diseases, and other endocrine disorders. On the day of oocyte retrieval following human chorionic gonadotropin triggering, follicular fluid of mature follicles (>14 mm) was pooled and centrifuged at 400 × g for 10 min. The pellet was resuspended and incubated with 0.1% hyaluronidase (Sigma, USA) at 37°C for 20 min, and then added with Ficoll-Paque (GE Healthcare, Sweden) for density gradient centrifugation at 600 × g for 10 min. Purified GCs were isolated from the interlayer phase and stored at -80°C until use. The study was approved by the Institutional Review Board of the First Affiliated Hospital of Wenzhou Medical University (No. 2021-08), and written informed consents were obtained from all participants.

### Real-time quantitative polymerase chain reaction

Total RNA was extracted from GC samples using TRIzol Reagent (Invitrogen, USA), and reverse transcribed into cDNA with SweScript RT I First Strand cDNA Synthesis Kit (Servicebio, China) after concentration and purity measurement by NanoDrop 2000 (Thermo Fisher Scientific, USA). RT-qPCR was performed in triplicates on the StepOnePlus Real-Time PCR System (Applied Biosystems, USA) using 2 × SYBR Green qPCR Master Mix (Servicebio, China). The mRNA expression level was quantified by the 2^-△△Ct^ method, with β-actin used as the internal reference gene for normalization. The primer sequences of target genes are listed in **Supplementary Table S2**.

### Assessment of oocyte quality

Fertilization check was performed 16-18 hours after insemination. Cleavage-stage embryos were graded on day 3 according to the Cummins’s morphological criteria (36). A total of 4 outcomes related to oocyte quality were assessed, including oocyte retrieval rate (oocytes retrieved out of ≥14 mm follicles on trigger day), mature oocyte rate (metaphase II oocytes out of oocytes retrieved), normal fertilization rate (two pronuclei oocytes out of oocytes retrieved), and good-quality embryo rate (day 3 embryos with grade I or II out of all day 3 embryos).

### Statistical analysis

For continuous variables, data were presented as mean ± standard deviation and assessed for normality using the Shapiro-Wilk test. Normally distributed data were compared by Student’s *t* test, while nonparametric data were compared by Mann-Whitney *U* test. Categorical variables were described as number with proportion, and Fisher’s exact test was used for comparison. Univariate correlations between core gene expression and oocyte competence parameters were calculated by the Spearman’s test. All statistical analyses were performed using SPSS software (version 26.0). A two-tailed *P*<0.05 was considered as statistically significant.

## Results

### Screening of ferroptosis-related DEGs

**Figure 1:**
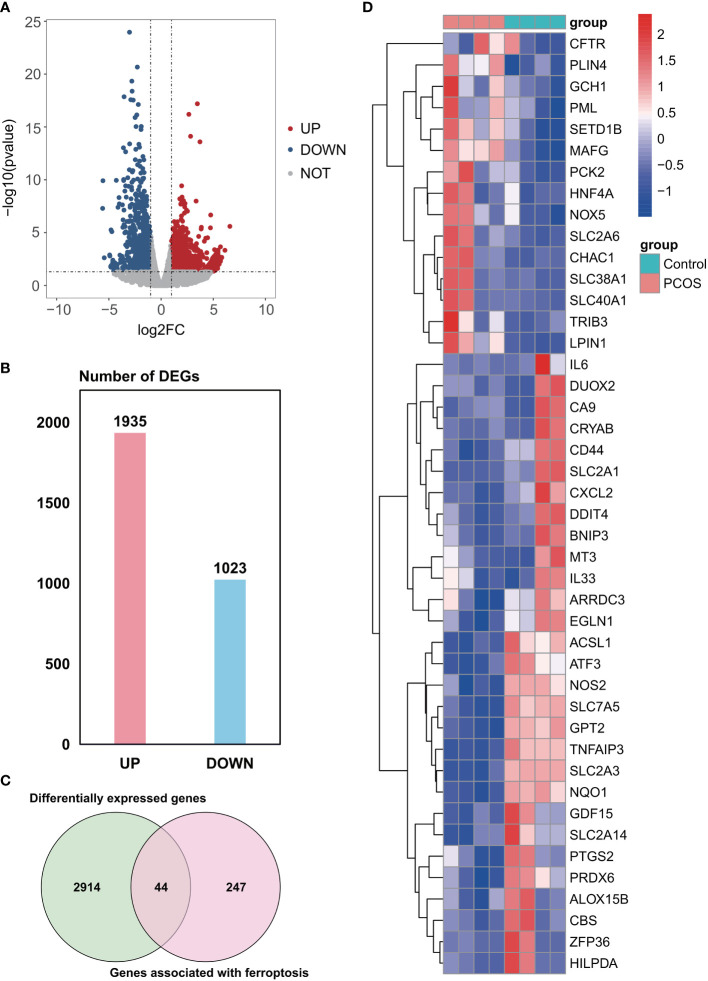
Identification of ferroptosis-related DEGs in the GSE155489 dataset. **(A)** Volcano plot of DEGs. **(B)** Number of DEGs. **(C)** Venn diagram showing the intersection between DEGs and genes associated with ferroptosis. **(D)** Clustered heatmap of ferroptosis-related DEGs in GSE155489. DEGs, differentially expressed genes.

**Figure 2:**
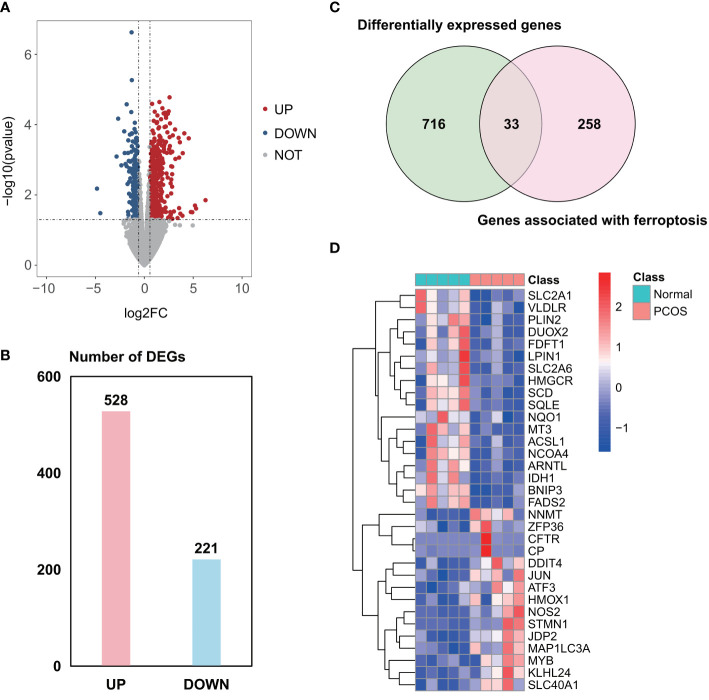
Identification of ferroptosis-related DEGs in the GSE168404 dataset. **(A)** Volcano plot of DEGs. **(B)** Number of DEGs. **(C)** Venn diagram showing the intersection between DEGs and genes associated with ferroptosis. **(D)** Clustered heatmap of ferroptosis-related DEGs in GSE168404. DEGs, differentially expressed genes.

A total of 2958 DEGs were obtained from the GSE155489 dataset, of which 1935 were upregulated and 1023 were downregulated in GCs of PCOS patients compared with control (**Figures 1A, B**). Among them, 44 genes were associated with ferroptosis as shown in clustered heatmap (**Figures 1C, D**). In the GSE168404 dataset, we isolated another 528 upregulated and 221 downregulated genes (**Figures 2A, B**), with 33 genes related to ferroptosis (**Figures 2C, D**). After intersection, 14 common ferroptosis-related DEGs were identified in both datasets (**Supplementary Figure S1**).

### Functional enrichment analysis

**Figure 3:**
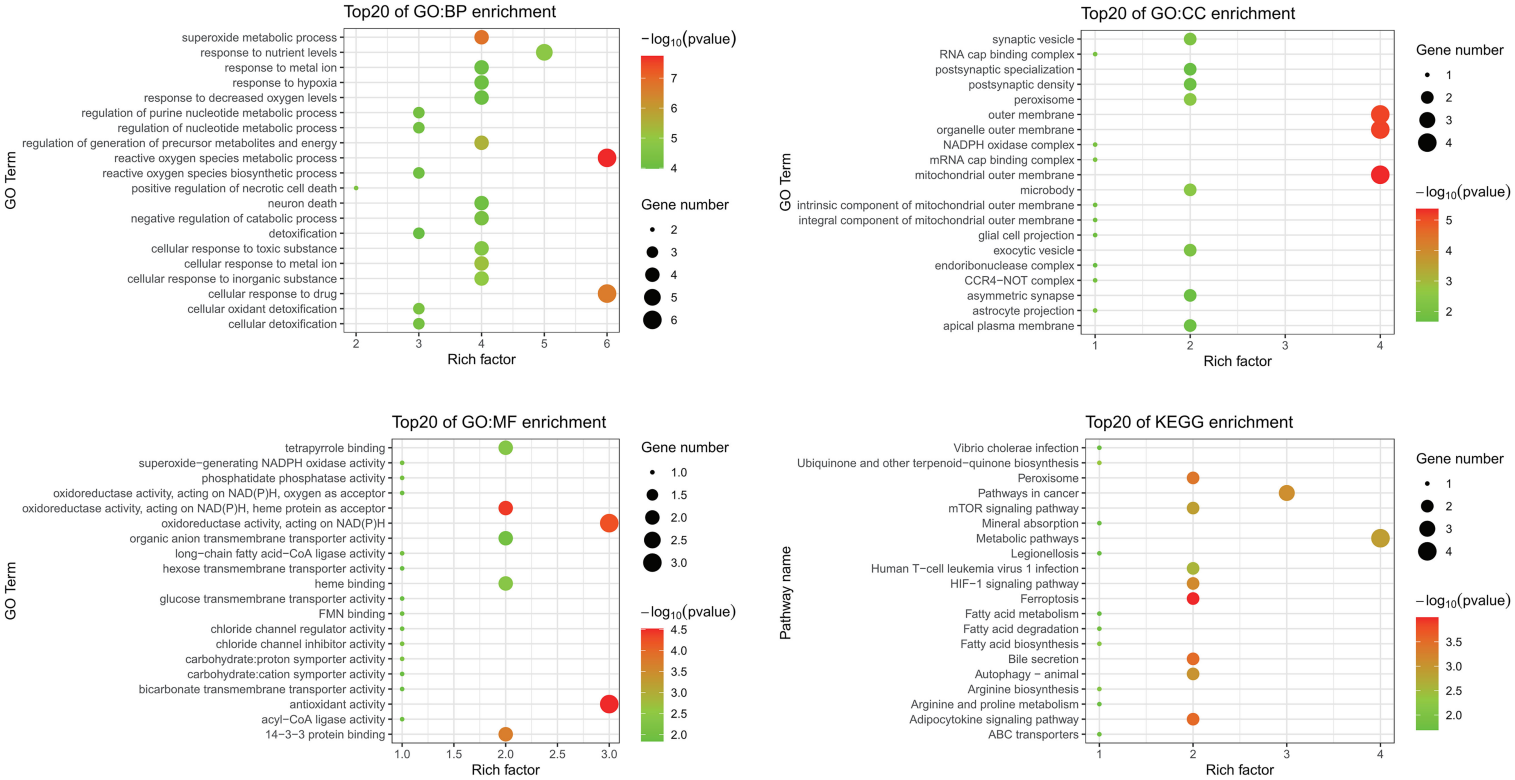
GO and KEGG enrichment analyses of ferroptosis-related DEGs. GO, Gene Ontology; KEGG, Kyoto Encyclopedia of Genes and Genomes; DEGs, differentially expressed genes; BP, biological process; CC, cellular component; MF, molecular function.

GO and KEGG enrichment analyses were performed to investigate functions and related pathways of the 14 candidate ferroptosis-related DEGs (**Figure 3**). In the GO analysis, genes were mainly enriched in reactive oxygen species metabolic process (GO:0072593) and superoxide metabolic process (GO:0006801) of the BP category; mitochondrial outer membrane (GO:0005741) and organelle outer membrane (GO:0031968) of the CC category; and antioxidant activity (GO:0016209) and oxidoreductase activity, acting on NAD(P)H (GO:0016651) of the MF category. KEGG analysis revealed that these genes mostly participated in ferroptosis (hsa04216), peroxisome (hsa04146), bile secretion (hsa04976), as well as adipocytokine signaling pathway (hsa04920).

### Identification of core ferroptosis-related DEGs

**Figure 4:**
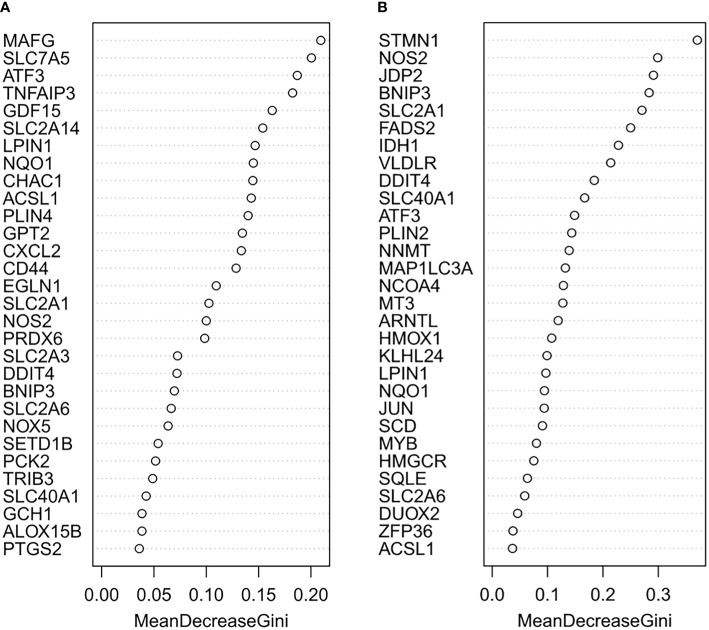
Random forest screening of core ferroptosis-related DEGs. **(A)** Mean Decrease Gini of top 30 ferroptosis-related DEGs in GSE155489. **(B)** Mean Decrease Gini of top 30 ferroptosis-related DEGs in GSE168404. DEGs, differentially expressed genes.

**Figure 5:**
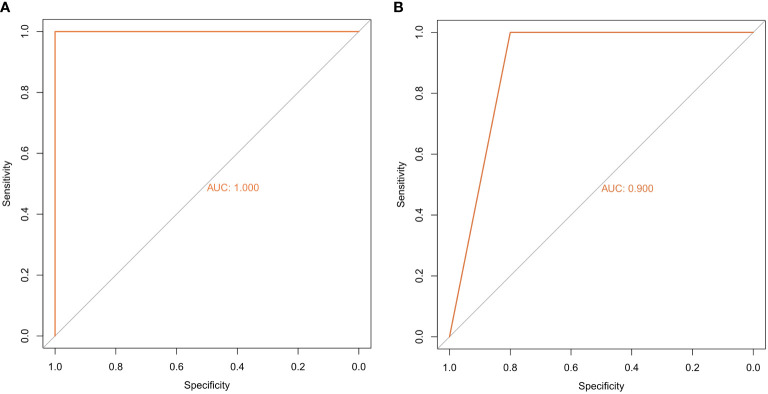
Diagnostic performance of core ferroptosis-related DEGs for polycystic ovary syndrome. **(A)** ROC curve of core ferroptosis-related DEGs in GSE155489. **(B)** ROC curve of core ferroptosis-related DEGs in GSE168404. DEGs, differentially expressed genes. ROC, receiver operating characteristic. AUC, area under the curve.

The random forest model was used to conduct deep learning on the sample data of GSE155489 and GSE168404. According to MDG of each ferroptosis-related DEGs, 43.2% (19/44) and 18.2% (6/33) of candidates with low importance (MDG<0.05) were filtered out respectively (**Figure 4**). By taking intersection of retaining genes in both datasets, 8 core genes were finally identified, including ATF3, BNIP3, DDIT4, LPIN1, NOS2, NQO1, SLC2A1 and SLC2A6 (**Supplementary Figure S1**). ROC curve analysis further demonstrated that a combination of these 8 genes had high diagnostic performance for PCOS, with an AUC of 1.000 in GSE155489 and 0.900 in GSE168404 (**Figure 5**).

### Construction of gene regulatory network

**Figure 6:**
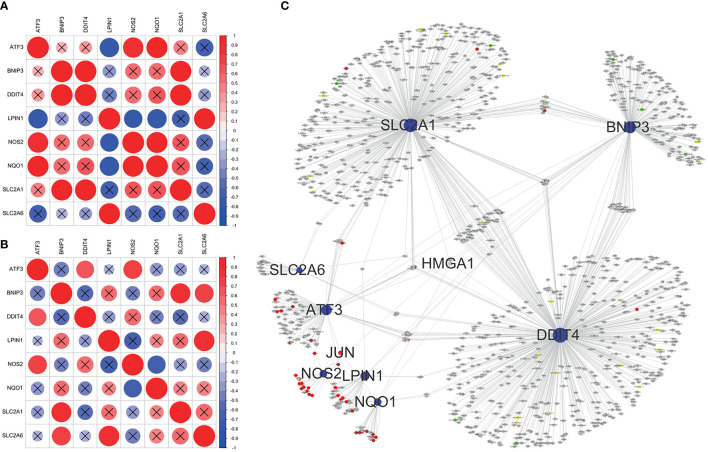
Correlation analysis and regulatory network of core ferroptosis-related DEGs. **(A)** Correlation heatmap of core ferroptosis-related DEGs in GSE155489. **(B)** Correlation heatmap of core ferroptosis-related DEGs in GSE168404. **(C)** Construction of gene regulatory network based on ENCORI and TRRUST databases. The blue nodes represent core ferroptosis-related DEGs, the red nodes represent transcription factors, the grey nodes represent messenger RNAs, the green nodes represent microRNAs, and the yellow nodes represent long non-coding RNAs. Nodes with degree ≥5 are shown with name. DEGs, differentially expressed genes.

Correlation analysis showed significant correlation among core ferroptosis-related DEGs in both datasets (**Figures 6A, B**), implying synergistic interaction and crosstalk of genes on expression. Subsequently, a regulatory network of core genes and their predicted mRNAs, miRNAs, lncRNAs, and TFs was generated on the grounds of ENCORI and TRRUST databases (**Figure 6C**). The results revealed JUN and HMGA1 as two potentially important TFs. Specifically, JUN could transcriptionally regulate the expression of ATF3, NOS2 and NQO1, and HMGA1 could regulate the expression of ATF3, BNIP3, DDIT4, NQO1 and SLC2A1.

### Prediction of potential therapeutic compounds

**Figure 7:**
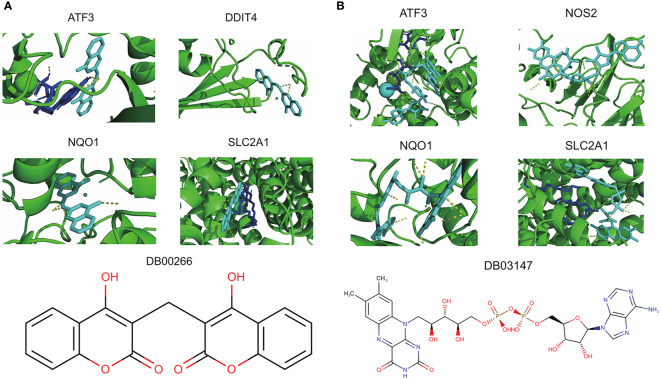
Potential therapeutic compounds corresponding to core ferroptosis-related DEGs. **(A)** Molecular docking of dicoumarol (DB00266) with ATF3, DDIT4, NQO1 and SLC2A1. **(B)** Molecular docking of flavin adenine dinucleotide (DB03147) with ATF3, NOS2, NQO1 and SLC2A1. DEGs, differentially expressed genes.

Of the 8 core genes, crystallographic protein structures of ATF3, DDIT4, NOS2, NQO1 and SLC2A1 were available in Protein Data Bank and were downloaded with code 4UYA, 3LQ9, 5XN3, 2F1O and 6THA, respectively. These proteins were then subjected for molecular docking with all compounds from DrugBank database. As a result, we identified dicoumarol (DB00266) and flavin adenine dinucleotide (DB03147) as two small molecules with therapeutic potential (**Figures 7A, B**). In detail, dicoumarol was docked with ATF3, DDIT4, NQO1 and SLC2A1, and flavin adenine dinucleotide had high binding affinity with ATF3, NOS2, NQO1 and SLC2A1 (**Supplementary Table S3**).

### Validation of core gene expression in GCs and association with oocyte quality

**Figure 8:**
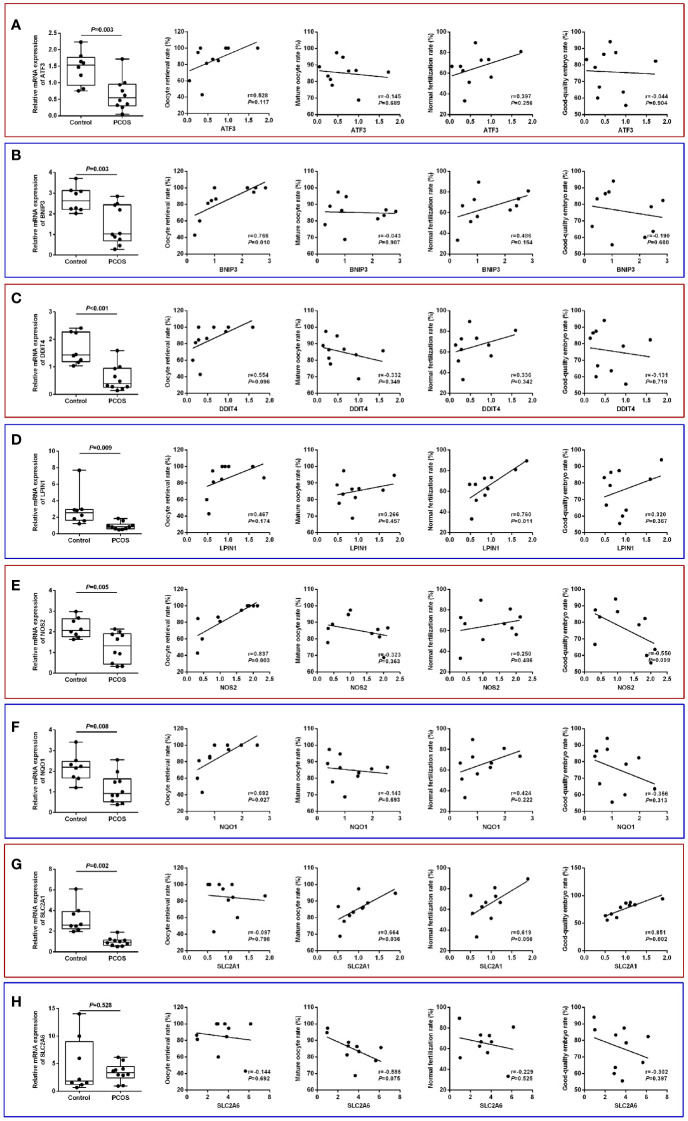
Validation of core ferroptosis-related DEGs in granulosa cells and association with oocyte quality. **(A)** ATF3. **(B)** BNIP3. **(C)** DDIT4. **(D)** LPIN1. **(E)** NOS2. **(F)** NQO1. **(G)** SLC2A1. **(H)** SLC2A6. DEGs, differentially expressed genes.

The identified core ferroptosis-related DEGs were further assayed by RT-qPCR on a sample set of 10 PCOS patients and 8 normo-ovulatory controls. There were no differences in age, body mass index, basal follicle stimulating hormone level, as well as infertility duration and type between the two populations. Contrarily, PCOS patients had significantly higher antral follicle count, anti-Müllerian hormone, basal luteinizing hormone, and total testosterone than controls (**Supplementary Table S4**). RT-qPCR results showed that the expression levels of 7 core genes, including ATF3, BNIP3, DDIT4, LPIN1, NOS2, NQO1 and SLC2A1, were significantly downregulated in GCs of PCOS patients, whereas no significant difference was detected in SLC2A6 (**Figures 8A–H**).

Comparison of IVF laboratory outcomes revealed a poorer oocyte competence in PCOS patients, as indicated by the lower oocyte retrieval rate (85.0 ± 19.5% vs. 94.6 ± 9.0%), mature oocyte rate (85.1 ± 8.2% vs. 92.3 ± 5.8%), normal fertilization rate (65.3 ± 15.8% vs. 72.4 ± 8.6%), and good-quality embryo rate (75.8 ± 13.3% vs. 85.1 ± 10.7%) relative to controls (**Supplementary Table S4**). In the PCOS group, the oocyte retrieval rate was significantly and positively correlated with BNIP3 (r=0.766, *P*=0.010), NOS2 (r=0.837, *P*=0.003), and NQO1 (r=0.692, *P*=0.027) mRNA levels, while normal fertilization rate was associated with LPIN1 (r=0.760, *P*=0.011) mRNA level. Additionally, the expression of SLC2A1 had significant correlations with both mature oocyte rate (r=0.664, *P*=0.036) and good-quality embryo rate (r=0.851, *P*=0.002) (**Figures 8A–H**).

## Discussion

Despite its prevalence in reproductive-aged women, the precise pathophysiology of PCOS remains incompletely elucidated. Ferroptosis is an iron-dependent and ROS-reliant regulated cell death, while both iron overload and increased ROS have been previously observed in PCOS patients (4, 5, 18, 19). In the present study, we applied an integrated bioinformatic and experimental approach to explore the unrecognized role of GC ferroptosis on PCOS development, thus providing novel biomarkers for its diagnosis, treatment, and clinical relevance with oocyte quality.

By *in silico* analysis of GSE155489 and GSE168404 datasets, we firstly identified 14 candidate ferroptosis-related genes in GCs of PCOS. GO analysis revealed that these DEGs may influence the ROS and superoxide metabolic processes by regulating antioxidant and oxidoreductase activities, which may be the potential mechanisms by which ferroptosis affects the pathogenesis of PCOS. KEGG analysis indicated enrichment in the ferroptosis and adipocytokine signaling pathway, suggesting that adipocytokines may exert crucial roles in mediating ferroptosis in PCOS. Interestingly, various adipocytokines, including adiponectin, apelin, chemerin, irisin, vaspin and leptin, have been found to be differentially secreted in follicular fluid and involved in GC dysfunction of PCOS patients (37). Recent studies have also linked adipocytokine with ferroptosis resistance of cancer (38), while their relationship in PCOS deserves future validation.

After random forest model construction, 8 out of the 14 DEGs were further selected as core ferroptosis-related genes for subsequent studies. Among them, NQO1 functions as a ferroptosis suppressor, ATF3 and LPIN1 serve as ferroptosis drivers, while BNIP3, DDIT4, NOS2, SLC2A1 and SLC2A6 are evidenced as ferroptosis markers for both suppressors and drivers in different biological circumstances (27). Unexpectedly, these genes were all downregulated in GC samples of PCOS patients, implying that the ferroptosis regulatory mechanisms in PCOS may be multivariable and complicated. Since the identified core genes are not ferroptosis-specific, it is also possible that they may mediate the crosstalk between ferroptosis and other cellular functions in PCOS development, such as proliferation, apoptosis and autophagy (16). Indeed, some core genes have been previously studied with inconsistent results. For example, Mlinar et al. (39) found that LPIN1 expression was decreased in adipose tissue of PCOS patients and associated with insulin resistance, while Nikolić et al. (40) detected increased LPIN1 paralleled with enhanced glucocorticoid signaling in dihydrotestosterone-induced PCOS rats. In terms of NOS2, one study related its overexpression in GCs with inflammatory ovulation defects of PCOS (15), while another study concluded that the reduced NOS2 transcripts could compromise endothelial and immune functions in PCOS via lowering nitric oxide concentration (41). Therefore, further studies are needed to clarify the specific regulatory function in GC ferroptosis via *in vivo* and *in vitro* models.

Poor oocyte quality remains one of the major obstacles encountered by PCOS patients during IVF treatment (3–6). Gene expressions in GCs reflect the characteristics of oocytes, thus providing a noninvasive approach to assess oocyte quality (42). In our study, the expression levels of 5 core ferroptosis-related genes were found to be significantly correlated with one or more parameters of oocyte quality. Among them, SLC1A1 has been priorly investigated, while the other 4 genes were reported as prognosis biomarkers in PCOS for the first time. Nevertheless, the study by Kim et al. (43) found that glucose transporters SLC2As 9, 11 and 12, rather than SLC2A1, were associated with oocyte competence including maturation rate, fertilization rate, and implantation rate. This contradictory finding could be possibly attributed to the use of *in vitro* maturation without ovarian stimulation for oocyte collection, contrary to the GnRH-ant protocol for IVF in current work.

On the basis of molecular docking, dicoumarol and flavin adenine dinucleotide were screened as two potential therapeutic compounds corresponding to core ferroptosis-related genes in PCOS. Dicoumarol is initially used as an anticoagulant for vascular thrombosis, while recent studies have also documented its gonad-safe anticancer, antimicrobial, and antiviral activities (44, 45). It can act as an inhibitor for NQO1 by competing with NAD(P)H, and targets Mrp-1 to suppress cellular glutathione export (45, 46). Flavin adenine dinucleotide is an indispensable auxiliary factor for the activity of several flavoproteins, which regulate ROS production, antioxidant defense, protein folding, and chromatin remodeling in living systems (47, 48). It can also stabilize the structure and decrease protease-mediated degradation of short-chain acyl-coenzyme A dehydrogenase (49). Accumulating studies have demonstrated its efficacy in metabolic disorders (49), malignant tumor (47, 48), and hypertensive vascular remodeling (50). However, whether these two compounds are applicable in PCOS treatment by targeting ferroptosis remains to be further explored.

Several limitations should be taken into account when interpreting the findings of this study. Firstly, the gene expression analysis was based directly on count matrix uploaded in GEO, which may cause result deviation due to the different RNA-seq data processing flows in the original reports (51). Secondly, given the heterogeneous nature of PCOS, the sample size for external validation was relatively small and may thus limit the power to detect statistical significances in comparison and correlation analyses. In this regard, prospective studies with larger cohort size need to be carried out in the future. Thirdly, mRNA transcripts and protein expressions are not consistently correlated because of various factors such as post transcription machinery. For proteomic analysis, Zhang et al. (52) collected endometrial samples from 33 PCOS and 7 control women, and also quantified 5 key proteins associated with ferroptosis. However, data were still lacking in GCs, and our identified DEGs were not validated at protein level. Finally, the precise regulatory mechanism of ferroptosis in PCOS development is unclear. A combination of molecular, cellular and animal experiments is required to validate the role of these identified core genes.

## Conclusions

In summary, this is the first comprehensive report to study the differential expression of ferroptosis-related genes in GCs of PCOS. Several core genes were identified and validated, which had high diagnostic performance for PCOS and significant correlation with oocyte quality. Our findings contribute to a better understanding on the potential role of ferroptosis in PCOS pathogenesis, and lay a theoretical foundation for the discovery of novel pharmacological therapy.

## Data availability statement

The datasets presented in this study can be found in online repositories. The names of the repository/repositories and accession number(s) can be found in the article/**Supplementary Material**.

## Ethics statement

The study was approved by the Institutional Review Board of the First Affiliated Hospital of Wenzhou Medical University (No. 2021-08). Written informed consents were obtained from all participants.

## Author contributions

JH: Funding acquisition, Methodology, Writing – original draft, Writing – review & editing. HF: Writing – review & editing, Conceptualization, Writing – original draft. CL: Writing – review & editing, Methodology. KY: Visualization, Writing – review & editing. CX: Data curation, Methodology, Writing – review & editing. SX: Data curation, Formal analysis, Writing – review & editing. SF: Formal analysis, Visualization, Writing – review & editing. SC: Data curation, Visualization, Writing – review & editing. BW: Data curation, Methodology, Writing – review & editing. YS: Conceptualization, Investigation, Writing – review & editing. BX: Formal analysis, Writing – review & editing. HY: Writing – review & editing. NW: Writing – review & editing. JZ: Funding acquisition, Writing – review & editing.

## Supplementary material

Supplementary Figure 1 Identification of common ferroptosis-related DEGs in the GSE155489 and GSE168404 datasets. **(A)** Venn diagram showing the intersection of ferroptosis-related DEGs before random forest screening. **(B)** Venn diagram showing the intersection of ferroptosis-related DEGs after random forest screening. DEGs, differentially expressed genes.
