# Supplementary figures and images for: Dysregulation of ferroptosis-related genes in granulosa cells associates with impaired oocyte quality in polycystic ovary syndrome
Source: Front Endocrinol (Lausanne). 2024 Feb 6;15:1346842. doi: 10.3389/fendo.2024.1346842 (PMC10882713; doi:10.3389/fendo.2024.1346842)

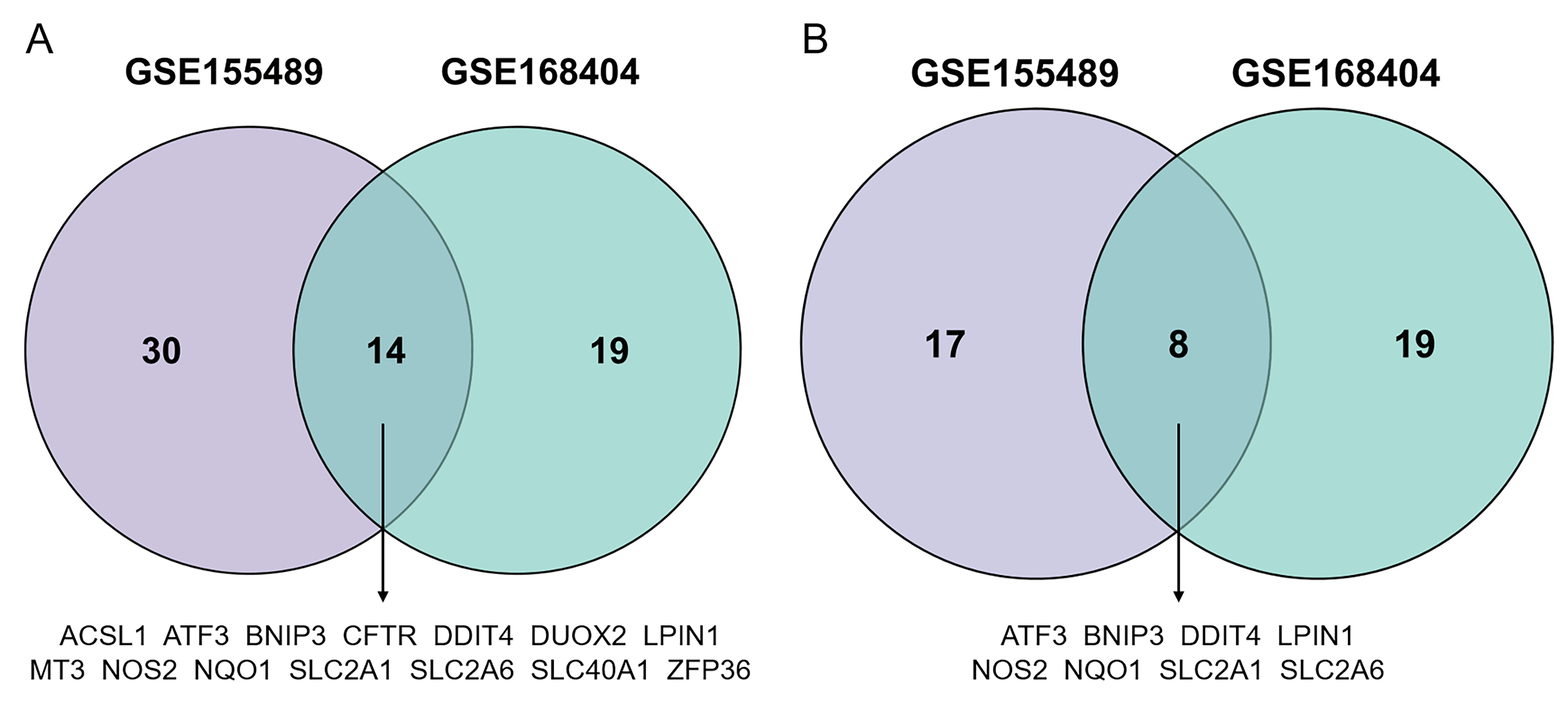

Supplement: Supplementary Figure 1 — Identification of common ferroptosis-related DEGs in the GSE155489 and GSE168404 datasets. (A) Venn diagram showing the intersection of ferroptosis-related DEGs before random forest screening. (B) Venn diagram showing the intersection of ferroptosis-related DEGs after random forest screening. DEGs, differentially expressed genes. [file Image_1.tiff]
