# Supplementary material for: Dysregulation of ferroptosis-related genes in granulosa cells associates with impaired oocyte quality in polycystic ovary syndrome
Source: Front Endocrinol (Lausanne). 2024 Feb 6;15:1346842. doi: 10.3389/fendo.2024.1346842 (PMC10882713; doi:10.3389/fendo.2024.1346842)
Supplement: Supplementary file 2 [file Table_1.docx]

**Table S1.** The merged ferroptosis-related gene set.

| Genes  (*n* = 291) | | | | | | | |
| --- | --- | --- | --- | --- | --- | --- | --- |
| ABCC1 | ATP6V1G2 | DUOX2 | HAMP | LPIN1 | NOS2 | SELENOS | TMBIM4 |
| ACACA | AURKA | DUSP1 | HBA1 | LURAP1L | NOX1 | SESN2 | TNFAIP3 |
| ACO1 | BACH1 | EGFR | HELLS | MAFG | NOX3 | SETD1B | TP53 |
| ACSF2 | BAP1 | EGLN1 | HERPUD1 | MAP1LC3A | NOX4 | SIRT1 | TP63 |
| ACSL1 | BECN1 | EGLN2 | HIC1 | MAP1LC3B | NOX5 | SLC11A2 | TRIB3 |
| ACSL3 | BID | EIF2AK4 | HIF1A | MAP1LC3C | NQO1 | SLC1A4 | TSC22D3 |
| ACSL4 | BLOC1S5-TXNDC5 | EIF2S1 | HILPDA | MAP3K5 | NRAS | SLC1A5 | TUBE1 |
| ACSL5 | BNIP3 | ELAVL1 | HMGB1 | MAPK1 | OTUB1 | SLC2A1 | TXNIP |
| ACSL6 | BRD4 | EMC2 | HMGCR | MAPK14 | OXSR1 | SLC2A12 | TXNRD1 |
| ACVR1B | CA9 | ENPP2 | HMOX1 | MAPK3 | PANX1 | SLC2A14 | UBC |
| AGPAT3 | CAPG | EPAS1 | HNF4A | MAPK8 | PCBP1 | SLC2A3 | ULK1 |
| AIFM2 | CARS | FADS2 | HRAS | MAPK9 | PCBP2 | SLC2A6 | ULK2 |
| AKR1C1 | CARS1 | FANCD2 | HSBP1 | MDM2 | PCK2 | SLC2A8 | VDAC2 |
| AKR1C2 | CAV1 | FBXW7 | HSD17B11 | MIF | PEBP1 | SLC38A1 | VDAC3 |
| AKR1C3 | CBS | FDFT1 | HSF1 | MIOX | PGD | SLC39A14 | VEGFA |
| ALB | CD44 | Fer1HCH | HSPA5 | MIR137 | PHKG2 | SLC39A8 | VLDLR |
| ALOX12 | CDKN1A | FH | HSPB1 | MIR17 | PIK3CA | SLC3A2 | WIPI1 |
| ALOX12B | CDKN2A | FLT3 | IDH1 | MIR212 | PLIN2 | SLC40A1 | WIPI2 |
| ALOX15 | CDO1 | FTH1 | IFNG | MIR30B | PLIN4 | SLC7A11 | XBP1 |
| ALOX15B | CEBPG | FTL | IL33 | MIR4715 | PML | SLC7A5 | YWHAE |
| ALOX5 | CFTR | FTMT | IL6 | MIR6852 | PRDX1 | SNORA16A | YY1AP1 |
| ALOXE3 | CHAC1 | G3BP1 | IREB2 | MIR9-1 | PRDX6 | SNX4 | ZEB1 |
| ANGPTL7 | CHMP5 | G6PD | ISCU | MIR9-2 | PRKAA1 | SOCS1 | ZFP36 |
| ANO6 | CHMP6 | G6PDX | ITGA6 | MIR9-3 | PRKAA2 | SP1 | ZFP69B |
| ARNTL | CISD1 | GABARAPL1 | JDP2 | MT1G | PRNP | SQLE | ZNF419 |
| ARRDC3 | CISD2 | GABARAPL2 | JUN | MT3 | PROM2 | SQSTM1 |  |
| ASNS | CP | GABPB1 | KEAP1 | MTDH | PSAT1 | SRC |  |
| ATF3 | CRYAB | GCH1 | KIM-1 | MTOR | PTGS2 | SRXN1 |  |
| ATF4 | CS | GCLC | KLHL24 | MUC1 | RB1 | STAT3 |  |
| ATG13 | CXCL2 | GCLM | KRAS | MYB | RELA | STEAP3 |  |
| ATG16L1 | CYBB | GDF15 | LAMP2 | MYC | RGS4 | STMN1 |  |
| ATG3 | DDIT3 | GLS2 | LINC00336 | NCF2 | RIPK1 | TAZ |  |
| ATG4D | DDIT4 | GLUT13 | LINC00472 | NCOA4 | RPL8 | TF |  |
| ATG5 | DNAJB6 | GOT1 | LOC284561 | NF2 | RRM2 | TFAP2C |  |
| ATG7 | DPP4 | GPT2 | LOC390705 | NFE2L2 | SAT1 | TFR2 |  |
| ATM | DRD4 | GPX2 | LONP1 | NFS1 | SAT2 | TFRC |  |
| ATP5G3 | DRD5 | GPX4 | LOX | NGB | SCD | TGFBR1 |  |
| ATP5MC3 | DUOX1 | GSS | LPCAT3 | NNMT | SCP2 | TLR4 |  |
