# Supplementary material for: Dysregulation of ferroptosis-related genes in granulosa cells associates with impaired oocyte quality in polycystic ovary syndrome
Source: Front Endocrinol (Lausanne). 2024 Feb 6;15:1346842. doi: 10.3389/fendo.2024.1346842 (PMC10882713; doi:10.3389/fendo.2024.1346842)
Supplement: Supplementary file 3 [file Table_2.docx]

**Table S2.** List of primers used for RT-qPCR.

| Genes | Primer forward (5’-3’) | Primer reverse (5’-3’) |
| --- | --- | --- |
| ATF3 | AAGTGAGTGCTTCTGCCATCG | CTCCTTCTTCTTGTTTCGGCAC |
| BNIP3 | CGCAGACACCACAAGATACCA | GCCGACTTGACCAATCCCATA |
| DDIT4 | CTTCTCGTCGTCGTCCACCT | CCATCCAGGTAAGCCGTGTCT |
| LPIN1 | GTTCCCGACCTTCAACACCTA | GGGCTGGACTCTTTCATCTTGT |
| NOS2 | TCGTGGAGACGGGAAAGAAG | CCTGGGTCCTCTGGTCAAACT |
| NQO1 | AAACTGAAGGACCCTGCGAAC | CAAACTCTCCTATGAACACTCGCT |
| SLC2A6 | TACACATCCCCTGTCATCCCA | GTCGTTGAGGATCATGGCACT |
| SLC2A1 | ATGGAGCCCAGCAGCAAGA | GGCATTGATGACTCCAGTGTTGTA |
| β-actin | CACCCAGCACAATGAAGATCAAGAT | CCAGTTTTTAAATCCTGAGTCAAGC |
