# Supplementary material for: Dysregulation of ferroptosis-related genes in granulosa cells associates with impaired oocyte quality in polycystic ovary syndrome
Source: Front Endocrinol (Lausanne). 2024 Feb 6;15:1346842. doi: 10.3389/fendo.2024.1346842 (PMC10882713; doi:10.3389/fendo.2024.1346842)
Supplement: Supplementary file 4 [file Table_3.docx]

**Table S3.** Molecular docking between potential therapeutic compounds and corresponding targets.

| DrugBank_ID | Protein | Affinity (kcal/mol) | rmsd l.b. | rmsd u.b. |
| --- | --- | --- | --- | --- |
| DB00266 | ATF3 | -7.2 | 0 | 0 |
|  | DDIT4 | -7.0 | 0 | 0 |
|  | NQO1 | -9.9 | 0 | 0 |
|  | SLC2A1 | -8.1 | 0 | 0 |
| DB03147 | ATF3 | -8.9 | 0 | 0 |
|  | NOS2 | -7.4 | 0 | 0 |
|  | NQO1 | -12.1 | 0 | 0 |
|  | SLC2A1 | -9.2 | 0 | 0 |
