# Supplementary material for: Dysregulation of ferroptosis-related genes in granulosa cells associates with impaired oocyte quality in polycystic ovary syndrome
Source: Front Endocrinol (Lausanne). 2024 Feb 6;15:1346842. doi: 10.3389/fendo.2024.1346842 (PMC10882713; doi:10.3389/fendo.2024.1346842)
Supplement: Supplementary file 5 [file Table_4.docx]

**Table S4.** Baseline demographics and clinical characteristics in PCOS and control patients.

|  | Control (*n* = 8) | PCOS (*n* = 10) | *P*-value |
| --- | --- | --- | --- |
| Age (years) | 31.5 ± 2.4 | 31.3 ± 1.6 | 0.833 ^a^ |
| Body mass index (kg/m^2^) | 24.7 ± 4.0 | 25.3 ± 3.7 | 0.717 ^a^ |
| Basal FSH (IU/L) | 7.5 ± 1.3 | 6.7 ± 2.2 | 0.237 ^b^ |
| Basal LH (IU/L) | 4.7 ± 1.4 | 8.7 ± 3.9 | 0.012 ^b^ |
| LH to FSH ratio | 0.64 ± 0.23 | 1.35 ± 0.68 | 0.003 ^b^ |
| Total testosterone (nmol/L) | 1.2 ± 0.4 | 2.2 ± 0.5 | <0.001 ^a^ |
| Anti-Müllerian hormone (ng/mL) | 4.4 ± 1.5 | 9.6 ± 5.7 | 0.004 ^b^ |
| Antral follicle count | 16.6 ± 2.6 | 31.9 ± 6.4 | <0.001 ^b^ |
| Infertility duration (years) | 3.7 ± 3.0 | 3.3 ± 2.1 | 0.714 ^a^ |
| Infertility type, *n* (%) |  |  | 0.637 ^c^ |
| Primary | 3 (37.5) | 6 (60) |  |
| Secondary | 5 (62.5) | 4 (40) |  |
| Stimulation duration (days) | 9.9 ± 1.4 | 9.3 ± 1.6 | 0.203 ^b^ |
| Total gonadotropin dose (IU) | 1889.1 ± 594.4 | 1587.5 ± 626.4 | 0.315 ^b^ |
| Oocyte retrieval rate (%) | 94.6 ± 9.0 | 85.0 ± 19.5 | 0.315 ^b^ |
| Mature oocyte rate (%) | 92.3 ± 5.8 | 85.1 ± 8.2 | 0.053 ^a^ |
| Normal fertilization rate (%) | 72.4 ± 8.6 | 65.3 ± 15.8 | 0.273 ^a^ |
| Good-quality embryo rate (%) | 85.1 ± 10.7 | 75.8 ± 13.3 | 0.130 ^a^ |

Data are presented as mean ± standard deviation or number (proportion). PCOS, polycystic ovary syndrome; FSH, follicle-stimulating hormone; LH, luteinizing hormone.

^a^ Student’s *t* test.

^b^ Mann-Whitney *U* test.

^c^ Fisher’s exact test.
